# Associations of Del 301-303 alpha2B-adrenoceptor gene polymorphism with central hemodynamic parameters in the northern Russian population

**DOI:** 10.1152/physiolgenomics.00071.2017

**Published:** 2017-12-06

**Authors:** Vladimir N. Melnikov, Victor I. Baranov, Irina Yu. Suvorova, Sergey G. Krivoschekov

**Affiliations:** Scientific Research Institute of Physiology and Basic Medicine, Novosibirsk, Russia

**Keywords:** ADRA2B, arterial stiffness, cardiac cycle, pulse wave analysis, SNP

## Abstract

The ADRA2B gene 301–303 I/D polymorphism is associated with various cardiovascular phenotypes. However, an association of genotypes with the timing structure of cardiac cycle remains unclear. The central hemodynamic parameters were assessed by pulse wave analysis in 63 residents of the Kola Peninsula (68 N) aged 27–65 yr. The genotypes were determined by PCR. The paired comparisons revealed that II genotype carriers had higher values of augmentation index (*P* = 0.014), ejection duration (*P* = 0.045), and lower SEVR (*P* = 0.035) than DD homozygotes. Multiple regression analysis adjusted for age, body mass index, heart rate, and blood pressure confirmed these results. Further sex stratified analysis showed that the associations existed only in men (*n* = 33) whereas in women (*n* = 30) the differences were suggestive (*P* < 0.1). It is concluded that in a northern Russian population men carrying I allele have stiffer arteries, shorter diastole duration, and impaired coronary perfusion and seem to be at higher risk for cardiovascular diseases than DD carriers.

## BACKGROUND

Vascular alpha2-adrenergic receptors (ADR) postsynaptically mediate physiological responses to endogenous catecholamines and cause arterial and venous vasoconstriction in skeletal muscles and in the heart (1). It is the B subtype of the family of three alpha2 receptors that is considered the key subtype determining vascular tone regulation and hence influencing the dynamic component of arterial stiffness. The majority of studies have evaluated an association of the allele frequencies of alpha2B-ADR gene 301–303 Del polymorphism with the prevalence of cardiovascular pathological phenotypes and circulatory events in various cohorts. Some of them have shown evidence of association for ischemic stroke, sudden cardiac death, nonfatal coronary events, and hereditary sick sinus node syndrome, whereas others have not. The homozygote for I allele of the gene is more frequent in diabetic patients with silent myocardial ischemia compared with those with angina, whereas D allele carriers are more prevalent than I ones among Malaysian hypertensives. The deletion genotype is associated with slower heart rate recovery after exercise compared with the insertion genotype.

Unlike the majority of genetic studies that have investigated or reviewed pressure amplitudes (3) or pulse wave velocity (2) as a measure of arterial compliance, this work emphasizes the timing parameters of cardiac cycle [ejection duration(ED), diastole duration], aorta-to-radial pulse pressure (PP) amplification (Ampl), augmentation index (AIx@75), and subendocardial viability ratio (SEVR). The latter is considered a surrogate measure of afterload myocardial perfusion, reflects a ratio of myocardial oxygen supply and demand, and was shown to be associated with vascular disease and fatal cardiac events (4).

The contribution of gene polymorphisms to the variance of arterial distensibility phenotypes has been addressed in many studies. Among them are those describing the influence of angiotensin-aldosterone genes, matrix metalloproteinase genes, endothelial cell-related genes, e.g., *NOS3*, beta-adrenergic receptor genes. The latter are known to mediate positive inotropic and chronotropic effects and are responsible for differences in cardiac autonomous nervous system functions.

In their turn, *ADRA2B* gene polymorphisms have been found to determine physiological parameters other than cardiovascular phenotypes but indirectly influencing them. This consideration illustrates the complexity of the genetic regulation of the parameters explored in the present study and explains the supposed weakness of effects.

## PHENOTYPE

### 

#### Cohort details.

We examined 63 apparently healthy Russian individuals of Caucasoid race (27–65 yr, 30 women) working at an underground loparite mine in the Kola Peninsula (northwestern Russia, 68°N) in December 2014. The participants were selected if they were normotensive or prehypertensive (<140/90 mmHg) and had no cardiovascular, respiratory or metabolic diseases. All provided written consent, and the Institute's Ethic Committee approved the study protocol.

#### Type of study.

This is a population-based observational study.

#### Cardiovascular phenotypes.

Arterial wave characteristics, aortic hemodynamics, and time pattern of cardiac cycle were assessed by applanation tonometry using the SphygmoCor Pulse Wave Analysis system (AtCor Medical). The mean blood pressure was obtained from an integration of the waveform. The pulse pressure amplification from the aorta to radial artery was calculated according to the formula Ampl = PP_radial_/PP_aortic_ × 100. As described elsewhere, higher AIx and lower Ampl indicate greater arterial stiffness (5). The left ventricular ED (percentage of the length of cardiac cycle) is the systolic time. Using the point of ED, the areas under the systolic and diastolic parts of the pulse curve are then calculated. The first parameter has been shown to relate to the contractile work of the heart and to its oxygen consumption. The diastolic part is associated with the time for coronary perfusion and characterizes energy supply. The ratio is termed as SEVR (%) and characterizes the balance between the energy supply and demand on the heart. Low SEVR has been associated with high cardiovascular risks and mortality in various populations (4).

#### Details of the SNP studied.

Genomic DNA was extracted from the blood using commercial kits (BioSilica, Novosibirsk, Russia) at the Genetic Laboratory at the Institute of Physiology and Basic Medicine. The 301–303 insertion/deletion polymorphism in *ADRA2B* gene (rs28365031) was detected by polymerase chain reaction followed by agarose gel electrophoresis.

#### Analysis model.

Individuals with homozygous and heterozygous haplotypes were grouped, and mean values ± SD for tonometric parameters were calculated. Using SPSS-19 software (IBM), we analyzed differences among genotype groups by the Kruskal-Wallis test since the data were not normally distributed in the studied sample. The post hoc Dunnett's T3 test was used for multiple comparisons. Multivariable linear regression models were created (GLM option) for genotype-dependent outcomes to adjust for the effects of potential confounding variables including age, body mass index (BMI), heart rate (HR), and aortic systolic blood pressure (SBP). A value of *P* ≤ 0.05 was considered statistically significant, but the value ≤0.1 was also indicated to note a suggestive effect.

## RESULTS

For both sexes, no significant differences in age, BMI, HR, and BPs were observed between haplotypes. Homozygous male carriers of I allele had higher aortic systolic (*P* = 0.039) and PP (*P* = 0.022), greater AIx value (*P* = 0.003), lower PP amplification (*P* = 0.013), and hence stiffer peripheral arteries compared with DD carriers (Supplemental Table S1). (The online version of this article contains supplemental material.) The higher ED indicates the shortened diastole, low SEVR, and therefore worse conditions for afterload myocardial perfusion. In women, the differences among genotypes for ED, AIx, and SEVR, though weak, are similar to those observed in men. For the entire sample, the differences remain significant for ED, AIx, and SEVR. For these variables, the paired comparisons revealed significant differences between II and DD genotype but not among ID and DD haplotypes. The SEVR value in the joint sex group varies across genotypes irrespective of major confounders (age, HR, BMI, and SBP) in the multiple regression model (Supplemental Table S2). The effect of haplotypes explains 12% of the total variation of SEVR and exceeds that of age and BMI.

## INTERPRETATION

Our results extend prior evidence by showing that the *ADRA2B* polymorphism not only affects the large arteries compliance (2, 3) but also determines timing parameters of cardiac cycle. It is now unclear whether the northern environment can select certain hemodynamic phenotypes and genotypes and whether place of birth as seasonally dependent factor can influence genotype-phenotype associations in epigenetic context. Exploring the same northern population we have recently reported a season-of-birth specificity of arterial stiffness in men.

### 

#### Limitations.

Our sample was middle-aged to elderly mining workers originated from and living in the North. This could potentially limit generalizability to individuals who are younger or of other professions and places of residence. Since the study is exploratory, additional investigations with larger sample size to confirm these findings are warranted.

## GRANTS

This study was financially supported by the Presidium of the Russian Academy of Sciences and conducted in the framework of the “Program of Basic Researches for the Development of the Russian Arctic.”

## DISCLOSURES

The authors declare that they have no competing interests.

## AUTHOR CONTRIBUTIONS

V. N. M. wrote and translated the draft, analyzed and interpreted data, performed applanation tonometry. V. I. B. searched and analyzed literature, collected blood samples. I. Y. S. performed medical inspection and prepared manuscript. S. G. K. organized fieldwork and developed design. All authors read and approved the final manuscript.

## Supplemental Data

Tables S1 and S2 Tables S1 and S2 - .docx (42.5 KB)
