# Supplementary material for: Associations of Del 301-303 alpha2B-adrenoceptor gene polymorphism with central hemodynamic parameters in the northern Russian population
Source: Physiol Genomics. 2017 Dec 6;50(2):100–1. doi: 10.1152/physiolgenomics.00071.2017 (PMC5867614; doi:10.1152/physiolgenomics.00071.2017)
Supplement: Tables S1 and S2 — docx (42.5 KB) [file supplemental-data.docx]

# SUPPLEMENTARY MATERIAL

## APPENDIX 1

Table 1. Physical characteristics and parameters of central hemodynamics and arterial stiffness in healthy individuals by sex and *ADRA2B* genotype

| Parameter | *ADRA2B* genotypes | | | p1 | p2 |
| --- | --- | --- | --- | --- | --- |
|  | II (1) | ID (2) | DD (3) |  |  |
| ***Men*** | ***N=7*** | ***N=19*** | ***N=7*** |  |  |
| Age, years | 46.2±4.6 | 43.7±13.8 | 45.4±5.4 | 0.192 |  |
| BMI, kg/m^2^ | 29.8±5.1 | 26.2±4.6 | 25.3±4.1 | 0.190 |  |
| HR, beat/min | 82.0±9.5 | 77.0±10.3 | 80.0±14.8 | 0.607 |  |
| bSBP, mmHg | 137.2±9.7 | 129.6±10.3 | 127.0±9.1 | 0.176 |  |
| bDBP, mmHg | 87.7±10.2 | 86.2±8.9 | 84.6±6.0 | 0.810 |  |
| bMBP, mmHg | 105.8±9.7 | 99.8±8.1 | 98.3±4.7 | 0.198 |  |
| bPP, mmHg | 49.5±7.2 | 43.5±12.0 | 42.4±11.6 | 0.463 |  |
| aSBP, mmHg | 126.3±10.1 | 115.8±10.0 | 111.6±6.0 | **0.023** | **0.048**(1-2); **0.039**(1-3) |
| aDBP, mmHg | 89.0±9.5 | 87.3±8.9 | 86.0±5.4 | 0.719 |  |
| aMBP, mmHg | 105.8±9.7 | 99.8±8.2 | 98.3±4.7 | 0.194 |  |
| aPP, mmHg | 37.3±6.7 | 28.5±9.9 | 25.6±6.0 | **0.041** | 0.078(1-2); **0.022**(1-3) |
| Ampl, % | 135.2±13.9 | 156.4±24.0 | 164.6±16.1 | **0.050** | **0.048**(1-2); **0.013**(1-3) |
| Tm, % | 28.4±2.6 | 25.1±2.3 | 24.7±2.7 | **0.028** | 0.074(1-2); 0.078(1-3) |
| ED, % | 40.3±4.8 | 35.0±3.2 | 35.9±3.7 | **0.029** | 0.081(1-2) |
| Tr, % | 19.4±2.8 | 18.4±3.2 | 19.3±3.1 | 0.682 |  |
| Aix@75 | 28.0±8.7 | 8.4±17.3 | 7.6±10.1 | **0.021** | **0.005**(1-2); **0.003**(1-3) |
| SEVR, % | 130.7±27.3 | 165.9±22.6 | 163.1±28.6 | **0.024** | 0.062(1-2) |
| ***Women*** | ***N=8*** | ***N=16*** | ***N=6*** |  |  |
| Age, years | 41.6±17.3 | 43.6±14.3 | 47.5±15.0 | 0.822 |  |
| BMI, kg/m^2^ | 28.2±8.9 | 29.2±4.9 | 24.2±0.6 | 0.354 |  |
| HR, beat/min | 80.5±9.1 | 72.1±9.1 | 75.3±6.3 | 0.110 |  |
| bSBP, mmHg | 126.5±16.9 | 127.0±16.8 | 128.5±8.2 | 0.979 |  |
| bDBP, mmHg | 84.8±11.0 | 81.3±9.1 | 86.5±10.0 | 0.534 |  |
| bMBP, mmHg | 100.5±12.8 | 98.7±10.9 | 101.8±9.1 | 0.861 |  |
| bPP, mmHg | 41.8±9.7 | 45.8±13.5 | 42.0±2.9 | 0.683 |  |
| aSBP, mmHg | 115.5±14.9 | 118.4±16.8 | 117.3±9.7 | 0.914 |  |
| aDBP, mmHg | 86.3±11.2 | 82.4±9.3 | 88.3±9.6 | 0.472 |  |
| aMBP, mmHg | 100.5±12.8 | 98.7±10.9 | 102.3±9.1 | 0.830 |  |
| aPP, mmHg | 29.3±7.5 | 36.0±13.4 | 29.0±5.6 | 0.300 |  |
| Ampl, % | 145.4±20.4 | 130.9±17.9 | 147.0±25.2 | 0.155 |  |
| Tm, % | 27.7±2.0 | 27.0±2.1 | 25.8±1.6 | 0.332 |  |
| ED, % | 38.5±3.4 | 36.9±3.2 | 35.3±1.5 | 0.087 | 0.078(1-3) |
| Tr, % | 19.0±2.7 | 16.1±2.7 | 16.9±2.7 | 0.065 | 0.082(1-2) |
| AI@75 | 22.5±11.0 | 27.6±10.6 | 17.0±12.6 | 0.087 | 0.065(2-3) |
| SEVR, % | 144.0±15.2 | 150.3±18.1 | 164.3±9.1 | 0.073 | **0.048**(1-3) |
| ***Both sexes*** | ***N=15*** | ***N=35*** | ***N=13*** |  |  |
| Age, years | 43.6±9.2 | 43.7±10.1 | 46.1±11.2 | 0.394 |  |
| BMI, kg/m^2^ | 28.8±7.3 | 27.6±4.9 | 24.9±3.2 | 0.321 |  |
| HR, beat/min | 81.1±8.9 | 74.8±9.9 | 78.2±12.2 | 0.154 |  |
| bSBP, mmHg | 131.1±14.9 | 128.4±13.5 | 127.6±8.4 | 0.812 |  |
| bDBP, mmHg | 86.0±10.3 | 83.9±9.2 | 85.3±7.0 | 0.579 |  |
| bMBP, mmHg | 102.8±11.5 | 99.3±9.4 | 99.6±6.4 | 0.519 |  |
| bPP, mmHg | 45.1±9.3 | 44.5±12.6 | 42.3±9.1 | 0.853 |  |
| aSBP, mmHg | 120.1±13.8 | 117.0±13.4 | 113.7±7.6 | 0.547 |  |
| aDBP, mmHg | 87.4±10.2 | 85.0±9.3 | 86.8±6.8 | 0.497 |  |
| aMBP, mmHg | 102.8±11.5 | 99.3±9.4 | 99.7±6.5 | 0.552 |  |
| aPP, mmHg | 32.7±8.1 | 31.9±12.0 | 26.8±5.8 | 0.308 |  |
| Ampl, % | 141.0±18.1 | 144.8±24.7 | 158.2±20.6 | 0.226 |  |
| Tm, % | 28.0±2.2 | 26.0±2.4 | 25.1±2.3 | **0.019** | **0.027**(1-2); **0.013**(1-3) |
| ED, % | 39.3±4.0 | 35.9±3.3 | 35.6±3.0 | **0.037** | **0.030**(1-2); **0.045**(1-3) |
| Tr, % | 19.2±2.7 | 17.4±3.2 | 18.4±3.1 | 0.298 |  |
| AI@75 | 24.9±10.1 | 18.0±15.0 | 11.0±11.4 | 0.077 | **0.014** (1-3) |
| SEVR, % | 138.3±23.1 | 158.7±23.2 | 163.6±22.7 | **0.043** | **0.035**(1-2); **0.029**(1-3) |

SBP, DBP, MBP, systolic, diastolic, and mean blood pressures; PP, pulse pressure; b, brachial; a, aortic; BMI, body mass index; Ampl., aorta-to-radial pulse pressure amplification; Tm, time to maximum systolic pressure in aortic root; HR, heart rate; ED, ejection duration; Tr, time to reflection; Aix@75, augmentation index; SEVR, subendocardial viability ratio. P1, Kruskal-Wallis test; P2, *post hoc* T3 Dunnett test. P-values in boldface are significant.

Table 2. Results of the univariate analysis of variance and multiple regression (GLM) between subendocardial viability ratio (dependent variable), age, SBP, HR, BMI (covariates) and *ADRA2B* haplotypes (fixed factor) in healthy men and women

| Source of variation | F | p | Partial Eta  squared | Standardized  β |
| --- | --- | --- | --- | --- |
| Corrected model | 15.71 | <0.001 <0.004 | 0.53 | – |
| Intercept | 138.5 | <0.001 | 0.72 | – |
| *ADRA2B* haplotype | 3.22 | 0.049 | 0.12 | 0.260 |
| Age | 2.40 | 0.127 | 0.04 | 0.045 |
| HR | 35.63 | <0.001 | 0.39 | –0.154 |
| SBP | 9.74 | 0.003 | 0.15 | –0.551 |
| BMI | 2.48 | 0.121 | 0.04 | –0.131 |

## APPENDIX 2

**Extra references**

1. **Brodde OE, Leineweber K.** Beta2-adrenoceptor gene polymorphisms. *Pharmacogenet Genomics 15(5): 267-75, 2005.*
2. **Chen QJ , Lu L, Jin C, Wang LJ, Zhang RY, Zhang Q,** [**Hu J**](https://www.ncbi.nlm.nih.gov/pubmed/?term=Hu%20J%5BAuthor%5D&cauthor=true&cauthor_uid=20692245)**, Yang ZK,** [**Shen WF**](https://www.ncbi.nlm.nih.gov/pubmed/?term=Shen%20WF%5BAuthor%5D&cauthor=true&cauthor_uid=20692245)**.** Insertion/insertion genotype of α(2B)-adrenergic receptor gene polymorphism is associated with silent myocardial ischemia in patients with type 2 diabetes mellitus. *Clin Biochem. 43(15): 1201-4, 2010.*
3. **Chernova AA, Nikulina SIu, Shul'man VA, Kukushkina TS, Voevoda MI, Maksimov VN.** [Polymorphisms of 2B-adrenergic receptor and endothelial NO-Synthase genes in genesis of the hereditary sick sinus node syndrome](https://www.ncbi.nlm.nih.gov/pubmed/21878072) *Kardiologiia 52(5): 20-4, 2012.*
4. ***Di Micco L,*** [***Salvi P***](https://www.ncbi.nlm.nih.gov/pubmed/?term=Salvi%20P%5BAuthor%5D&cauthor=true&cauthor_uid=23735512)***,*** [***Bellasi A***](https://www.ncbi.nlm.nih.gov/pubmed/?term=Bellasi%20A%5BAuthor%5D&cauthor=true&cauthor_uid=23735512)***,*** [***Sirico ML***](https://www.ncbi.nlm.nih.gov/pubmed/?term=Sirico%20ML%5BAuthor%5D&cauthor=true&cauthor_uid=23735512)***,*** [***Di Iorio B***](https://www.ncbi.nlm.nih.gov/pubmed/?term=Di%20Iorio%20B%5BAuthor%5D&cauthor=true&cauthor_uid=23735512)*.* Subendocardial viability ratio predicts cardiovascular mortality in chronic kidney disease patients. [*Blood Purif*](https://www.ncbi.nlm.nih.gov/pubmed/?term=micco%2C+salvi%2C+bellasi) *36(1): 26-8, 2013.*
5. **Fava C, Montagnana M, Guerriero M, Almgren P, von Wowern F, Minuz P, Melander O.** Chromosome 2q12, the ADRA2B I/D polymorphism and metabolic syndrome. J Hypertens 27(9): 1794-803, 2009.
6. **Hashimoto J, Ito S.** Pulse pressure amplification, arterial stiffness, and peripheral wave reflection determine pulsatile flow waveform of the femoral artery. *Hypertension* 56(5): 926–33, 2010.
7. **Hein L, Altman JD, Kobilka BK.** Two functionally distinct alpha2-adrenergic receptors regulate sympathetic neurotransmission. *Nature 402(6758): 181-4, 1999.*
8. **Hoffman JI, Buckberg GD.** The myocardial oxygen supply:demand index revisited. *J Am Heart Assoc 3(1): e000285, 2014.*
9. **Kohli U, Diedrich A, Kannankeril PJ, Muszkat M, Sofowora GG, Hahn MK, et al.** Genetic variation in alpha2-adrenoceptors and heart rate recovery after exercise. *Physiol Genomics 47(9):400-6, 2015.*
10. **Lacolley P, Challande P, Osborne-Pellegrin M, Regnault V.** [Genetics and pathophysiology of arterial stiffness.](https://www.ncbi.nlm.nih.gov/pubmed/19098299) *Cardiovasc Res 81(4): 637-48, 2009.*
11. **Laukkanen JA, Mäkikallio TH, Kauhanen J, Kurl S.** Insertion/deletion polymorphism in alpha2-adrenergic receptor gene is a genetic risk factor for sudden cardiac death. *Am Heart J 158(4): 615-21, 2009.*
12. **Li LH, Li Y, Wen Y, Wang JG.** Anthropometric and metabolic phenotypes in relation to the in relation to the ADRA2B deletion/insertion polymorphysm in Chinese population. *J Hypertens 26(11): 2161-7, 2008.*
13. **Matsunaga T, Yasuda K, Adachi T, Gu N, Yamamura T, Moritani T, Tsujimoto G, Tsuda K.** Association of beta-adrenoceptor polymorphisms with cardiac autonomic modulation in Japanese males. *Am Heart J 154(4):759-66, 2007.*
14. **Melnikov V.N., Suvorova I.Yu., Belisheva N.K.** [Central hemodynamics and arterial stiffness in adult humans depend on the conditions of early development in the northern Kola Peninsula.] *Fiziol Cheloveka 42(2): 43-8, 2016 (In Russian. Eng. translation: Hum Physiol 42(2): 150-5, 2016.)*
15. **Oh SH, Min KT,** [**Jeon YJ**](https://www.ncbi.nlm.nih.gov/pubmed/?term=Jeon%20YJ%5BAuthor%5D&cauthor=true&cauthor_uid=22560155)**, Kim MH, Kim OJ, Shin BS, Oh D,** [**Kim NK**](https://www.ncbi.nlm.nih.gov/pubmed/?term=Kim%20NK%5BAuthor%5D&cauthor=true&cauthor_uid=22560155)**.** Association between common genetic variants of α2A-, α2B-, and α2C-adrenergic receptors and ischemic stroke. *Clin Neurol Neurosurg 115(1): 26-31, 2013.*
16. **Snapir A, Heinonen P, Tuomainen TP, Alhopuro P, Karvonen MK, Lakka TA, Nyyssönen K, Salonen R, Kauhanen J, Valkonen VP, Pesonen U, Koulu M, Scheinin M, Salonen JT.** An insertion/deletion polymorphism in the alpha2B-adrenergic receptor gene is a novel genetic risk factor for acute coronary events. *J Am Coll Cardiol 37(6): 1516-22, 2001.*
17. **Talke P, Stapelfeldt C, Lobo E, Brown R, Scheinin M, Snapir A.** Effect of alpha2B-adrenoceptor polymorphism on peripheral vasoconstriction in healthy volunteers. *Anesthesiology 102(3): 536-42, 2005.*
18. **Vasudevan R, Ismail P, Stanslas J, Shamsudin N, Ali AB.** Association of insertion/deletion polymorphism of alpha-adrenoceptor gene in essential hypertention with or without type 2 diabetes mellitus in Malaysian subjects. *Int J Biol Sci 4(6): 362-7, 2008.*
19. **Wilkinson IB, Mahammad NH, Tyrrell S, Hall IR, Webb DJ, Paul VE , Levy T, Cockcroft JR.** Heart rate dependency of pulse pressure amplification and arterial stiffness. *Am J Hypertens 15(1 Pt 1): 24–30, 2002.*
20. **Xia K, Ding R, Zhang Z, Li W, Shang X, Yang X, Wang L, Zhang Q.** The association of eight potentially functional polymorphisms in five adrenergic receptor-encoding genes with myocardial infarction risk in Han Chinese. *Gene 624: 43–9, 2017.*
21. [**Zhang HF**](https://www.ncbi.nlm.nih.gov/pubmed/?term=Zhang%20HF%5BAuthor%5D&cauthor=true&cauthor_uid=16336817)**,** [**Li XL**](https://www.ncbi.nlm.nih.gov/pubmed/?term=Li%20XL%5BAuthor%5D&cauthor=true&cauthor_uid=16336817)**,** [**Xie SF**](https://www.ncbi.nlm.nih.gov/pubmed/?term=Xie%20SF%5BAuthor%5D&cauthor=true&cauthor_uid=16336817)**,** [**Zhu J**](https://www.ncbi.nlm.nih.gov/pubmed/?term=Zhu%20J%5BAuthor%5D&cauthor=true&cauthor_uid=16336817)**,** [**Wang ZZ**](https://www.ncbi.nlm.nih.gov/pubmed/?term=Wang%20ZZ%5BAuthor%5D&cauthor=true&cauthor_uid=16336817)**,** [**Liang LR**](https://www.ncbi.nlm.nih.gov/pubmed/?term=Liang%20LR%5BAuthor%5D&cauthor=true&cauthor_uid=16336817)**,** [**Cao KJ**](https://www.ncbi.nlm.nih.gov/pubmed/?term=Cao%20KJ%5BAuthor%5D&cauthor=true&cauthor_uid=16336817)**,** [**De W**](https://www.ncbi.nlm.nih.gov/pubmed/?term=De%20W%5BAuthor%5D&cauthor=true&cauthor_uid=16336817)**,** [**Yuan L**](https://www.ncbi.nlm.nih.gov/pubmed/?term=Yuan%20L%5BAuthor%5D&cauthor=true&cauthor_uid=16336817)**,** [**Huang J**](https://www.ncbi.nlm.nih.gov/pubmed/?term=Huang%20J%5BAuthor%5D&cauthor=true&cauthor_uid=16336817)**.** ADRA2B gene insertion/deletion polymorphism and artery compliance. *Chin Med J (Engl) 118(21): 1797-802, 2005.*
